# Neurodegenerative Disease Related Proteins Have Negative Effects on SNARE-Mediated Membrane Fusion in Pathological Confirmation

**DOI:** 10.3389/fnmol.2017.00066

**Published:** 2017-03-21

**Authors:** Chen Hou, Yongyao Wang, Jiankang Liu, Changhe Wang, Jiangang Long

**Affiliations:** Center for Mitochondrial Biology and Medicine and Key Laboratory of Biomedical Information Engineering of the Ministry of Education, School of Life Science and Technology, and Frontier Institute of Science and Technology, Xi'an Jiaotong UniversityXi'an, China

**Keywords:** neurotransmitter release, membrane fusion, neurodegenerative diseases, single molecule biophysics technology, SNARE

Studies showed that synapses are highly-specialized structures for the communication between pre- and postsynaptic neurons (Kaeser and Regehr, 2014). Synaptic vesicles carrying neurotransmitters dock at specialized sites of presynaptic membranes termed active zones, which are closely apposed to postsynaptic densities, and then undergo one or more priming reactions to prepare them to a release-ready state. When an action potential invades the nerve terminals, the following membrane depolarization activates voltage-gated calcium channels to influx Ca^2+^, thus initiates the fusion of synaptic vesicles with presynaptic membrane and transmitters release (Wu and Saggau, 1997).

It was reported that synaptic vesicle fusion requires assembly of a conserved proteins family termed soluble N-ethylmaleimide-sensitive factor attachment protein receptors (SNAREs) (Chernomordik and Kozlov, 2008; Wickner and Schekman, 2008). All SNAREs contain an evolutionarily conserved coiled-coil SNARE motif of ~60–70 amino acids that are arranged in heptad repeats. In synapses, syntaxin and synaptosome-associated protein of 25 kDa (SNAP-25, contains two SNARE motifs) on the plasma membrane (t-SNAREs on target membrane) and synaptobrevin/vesicle-associated membrane protein (VAMP) on synaptic vesicles (v-SNARE) assemble into a tight *trans*-SNARE complex in a 1:1:1 ratio to bridge synaptic vesicles and the plasma membrane (Brunger, 2005). The *trans*-SNARE complex promotes membrane fusion by pulling the bilayers together as it zippers up, and the remaining SNARE complexes on the fused membrane are transformed to cis-configuration with lower potential energy, which undergoes disassembly catalyzed by a specialized adenosine triphosphatase (ATPase) *N*-ethylmaleimide-sensitive factor (NSF) and its cofactors soluble NSF attachment proteins (SNAPs) (Jahn et al., 2003). SNAPs bind directly to the SNARE complex, then recruit and activate NSF to completely dissociate SNARE complex and recycle individual SNAREs for a new round of fusion reactions (Sudhof and Rothman, 2009). Thus, it appears that the cycle of SNARE assembly and disassembly is critical for the occurrence, the fidelity and plasticity of synaptic transmission.

Meanwhile, it has been shown that a wide range of neurodegenerative disorders are characterized with neuronal dysfunction and neuron loss, which caused by the aggregation of specific neurotoxic proteins (Caughey and Lansbury, 2003). Typically, α-synuclein (α-syn), constitutes the amyloid fibril form of Lewy bodies (Wang et al., 2016), is a cytosolic neural protein consisting of 140 amino acid residues and is abundantly expressed in presynaptic membrane in monomeric form (Burre et al., 2013). α-syn is closely associated with early-onset of neurodegenerative diseases prominently in familial Parkinson's disease, Alzheimer's disease and Lewy body disease. Aβ, a peptide of 36–43 amino acids, is a key molecule in the pathogenesis of Alzheimer's disease, and Aβ deposition is the necessary prerequisites for synaptic dysfunction and cognitive impairment in Alzheimer's disease (Annaert and De Strooper, 2002). Studies showed that, in neurodegenerative diseases, the neurotransmission was profoundly damaged, and the SNARE protein function and distribution were changed (Garcia-Reitbock et al., 2010; Shen, 2010). Thus, we propose that these proteins in pathological confirmation play roles in SNARE-mediated membrane fusion during neurotransmission by disrupting the SNARE protein assembling and recycling.

Recent research showed that α-syn can be classified into spiral membrane binding form, part of folding state, oligomers, fibrous, and amorphous polymers, etc according to the molecular formation after polymerization (Breydo et al., 2012). It has been verified that α-syn oligomerization contributes to the increased cytotoxicity and thus promotes the dopaminergic neuronal degeneration (Hogen et al., 2012). However, native α-syn shows no damage effect on the efficiency of synaptic vesicle exocytosis, but helps to increase the availability of synthetic vesicles at the synapse (Diao et al., 2013a). In addition, α-syn knockout shows little effect on synaptic transmission (Nemani et al., 2010), while overexpression of α-syn reduces neurotransmitter release by disturbing vesicle docking in exocytosis (Larsen et al., 2006). The possible mechanisms may be due to the reduction of synaptic vesicle recycling pool size, the reduced synaptic vesicle density at the active zone and the defects in re-clustering of synaptic vesicles upon α-syn overexpression. Moreover, exhibiting the supportive role in the folding/refolding of SNARE proteins, study showed that α-syn acts as a non-classical chaperone that facilitates the maintenance of proper SNARE states during SNARE cycle, and promotes SNARE complex assembly by directly binding to synaptobrevin-2/VAMP2 (Burre et al., 2010). For example, monomeric α-syn contributes to neural vesicle aggregation by simultaneously interacting with synaptobrevin-2 (Diao et al., 2013a), however, α-syn oligomers inhibits vesicle docking through interaction with synaptobrevin-2 and negatively charged phospholipids (Choi et al., 2013; Hu et al., 2016). Therefore, it appears that the effects of α-syn on neurotransmission are mainly determined by forms of α-syn polymerization.

Cysteine string protein α (CSPα), a co-chaperone protein, also plays an important role in maintaining SNARE rapid cycling and neuronal activity (Garcia-Junco-Clemente et al., 2010). There were several reports that CSPα expression is greatly decreased in the forebrain from patients with neurodegenerative disorders (Tiwari et al., 2015). It was also shown that CSPα can form a chaperone complex with Hsc70 (Chamberlain and Burgoyne, 1997) and SGT protein (Nemani et al., 2010), and the CSPα–Hsc70–SGT complex binds directly to monomeric SNAP-25 to prevent its polymerization, enabling SNARE complex formation. Consistently, CSPα knockout mice show defects in synaptic function that associated with the abnormal formation of SNAP-25 (Fernandez-Chacon et al., 2004). In contrast, overexpression of CSP suppresses the degradation of SNAP-25 under normal physiological condition (Sharma et al., 2011). Dysfunctional SNAP-25, in the absence of CSPα, is ubiquitinated and degraded by the proteasome in a synaptic activity–dependent manner, leading to the reduction of SNAP-25 (Sharma et al., 2011). In addition, overexpression of α-syn blocks the CSPα deletion-induced neurodegeneration and ameliorates the CSPα deficiency-induced inhibition of SNARE complex assembly, however, the removal of endogenous α-syn deteriorated CSPα deletion-induced symptoms (Chandra et al., 2005). These phenomena imply that α-syn may cooperate with CSPα to maintain SNARE proteins assembly and neurotransmission.

Furthermore, it has been shown that intracellular Aβ oligomers inhibit SNARE-mediated exocytosis by impairing SNARE complex formation through direct interaction with syntaxin 1a (Yang et al., 2015). Aβ42 is reported to regulate neurotransmitter release, probably by disrupting the complex formation of Synaptophysin and VAMP2 through the competitive interaction with Synaptophysin (Russell et al., 2012). Studies also showed that Aβ oligomers contribute to the down-regulation of synapse density and decreased neurotransmission efficiency (Terry et al., 1991; Shankar et al., 2007). In addition, although aggregations of Aβ and α-syn are used as the major pathological markers of AD and PD respectively, these two pathogenic proteins have synergistic effects on neurodegenerative disorders (Choi et al., 2015). Study showed that Aβ promotes the formation of large-size α-syn oligomers, which function to inhibit SNARE-mediated vesicle fusion, accelerate motor or memory deficits or cognitive dysfunction in APP/PS1 transgenic mice (Yang et al., 2015). It suggested that these two proteins may cooperate to suppress membrane fusion, and that the formation of α-syn aggregates requires the participation of Aβ. Meanwhile, some other pathological proteins in Alzheimer's disease such as amyloid precursor protein, presenilin, phosphorylated tau protein, and brain-derived neurotrophic factor are associated with Aβ deposition and contribute to the regulation of neuronal function in Alzheimer's disease (Saura et al., 2004; Schindowski et al., 2008; Peethumnongsin et al., 2010).

The evidences above support the suggestion that these typical neurotoxic proteins have negative effects on the assembly and disassembly cycle of SNARE proteins, and thus on the SNARE-mediated membrane fusion during neurotransmission. In can be implied that SNARE-mediated membrane fusion in a functional state is largely depends on molecular chaperone systems, which is exhibited by α-syn, CSPα, Aβ, etc (Figure 1). These proteins directly or indirectly interact with one or more components of SNARE complex, chaperoning and maintaining appropriate SNARE protein complex assembly or disassembly under different pathological conditions.

**Figure 1:**
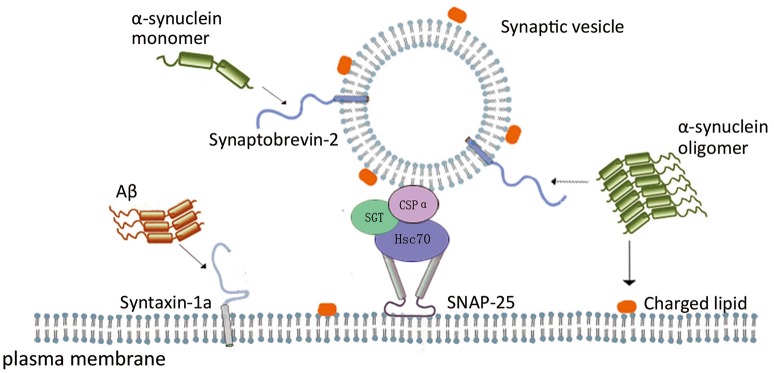
**Scheme of the role of neurodegenerative disease related proteins in SNARE-mediated membrane fusion**. In SNARE-mediated membrane fusion, α-syn monomer interacting with synaptobrevin-2, and α-syn oligomers inhibits vesicle docking through interaction with synaptobrevin-2 and negatively charged phospholipids. CSPα form a complex with Hsc70 and SGT protein, and the complex binds to monomeric SNAP-25 to prevent its polymerization, enabling SNARE complex formation. Aβ oligomers impair SNARE complex formation through interaction with syntaxin 1a.

It was also shown that the mechanism of SNARE-mediated membrane fusion during neurotransmission has been analyzed by real time monitoring SNARE protein interactions with single-molecule FRET (smFRET) imaging (Weninger et al., 2003, 2008; Bowen et al., 2004, 2005). There are two major smFRET assays applied for SNARE-mediated membrane fusion: Fusion proteins monitoring during fusion processes (Brunger et al., 2009) and the lipid molecule mixing between of the fused vesicles (Diao et al., 2009, 2012). In monitoring proteins, SNARE and its accessory proteins that are site-specifically conjugated with fluorescent dyes may be used to analyze the unique protein structural information (Brunger et al., 2009). In monitoring fusion, the FRET efficiency value from each pair of vesicles may be collected to identify different stages of fusion, such as docking, hemifusion, and full fusion (Diao et al., 2013b). These supports the suggestion that smFRET approach allows the detection down to the conformation of a single biomolecule with two dyes attached, and that smFRET approach is different from the ensemble fusion assay that averaged FRET signal from the entire population is obtained.

Finally, mechanistic studies of neurotransmitter release contribute significantly to clarify the pathogenesis of neurodegenerative diseases in brain, which will help to illuminate the underlying pathogenic mechanisms for neurodegenerative disorders. In recent years, the effects of these neurotoxic proteins at different stages of SNARE-mediated membrane fusion have been extensively investigated by single molecule biophysics technologies, including smFRET imaging (Brunger et al., 2015). SmFRET techniques will certainly benefit further studies about SNARE-mediated membrane fusion, and benefit the cross-talk investigation between neurodegenerative proteins and SNARE cycling during neurodegeneration. We suggest that future research should be integrated to the real-time monitoring of membrane fusion of neurotransmitter release *in vivo* (Sakon and Weninger, 2010; Crawford et al., 2013). This helps for the deeper understanding of neural signaling process and the exploration of new treatment for neurological disorders.

## Author contributions

CH drafted the manuscript; YW drawed the cartoon; JLi drafted the manuscript; CW revised the manuscript; JLo revised the manuscript.

## Funding

This work was supported by the National Basic Research Program of China (973 Program, 2015CB856302 and 2015CB553602), the opening foundation of the State Key Laboratory of Space Medicine Fundamentals and Application, the Chinese Astronaut Research and Training Center (SMFA15K01), and the National Natural Science Foundation of China (31400708 and 31670843).

### Conflict of interest statement

The authors declare that the research was conducted in the absence of any commercial or financial relationships that could be construed as a potential conflict of interest.

## References

[B1] Annaert W. De Strooper B. (2002). A cell biological perspective on Alzheimer's disease. Annu. Rev. Cell Dev. Biol. 18, 25–51. 10.1146/annurev.cellbio.18.020402.142302 12142279

[B2] Bowen M. E. Weninger K. Brunger A. T. Chu S. (2004). Single molecule observation of liposome-bilayer fusion thermally induced by soluble N-ethyl maleimide sensitive-factor attachment protein receptors (SNAREs). Biophys. J. 87, 3569–3584. 10.1529/biophysj.104.048637 15347585 PMC1304822

[B3] Bowen M. E. Weninger K. Ernst J. Chu S. Brunger A. T. (2005). Single-molecule studies of synaptotagmin and complexin binding to the SNARE complex. Biophys. J. 89, 690–702. 10.1529/biophysj.104.054064 15821166 PMC1366567

[B4] Breydo L. Wu J. W. Uversky V. N. (2012). Alpha-synuclein misfolding and Parkinson's disease. Biochim. Biophys. Acta 1822, 261–285. 10.1016/j.bbadis.2011.10.002 22024360

[B5] Brunger A. T. (2005). Structure and function of SNARE and SNARE-interacting proteins. Q. Rev. Biophys. 38, 1–47. 10.1017/S0033583505004051 16336742

[B6] Brunger A. T. Cipriano D. J. Diao J. (2015). Towards reconstitution of membrane fusion mediated by SNAREs and other synaptic proteins. Crit. Rev. Biochem. Mol. Biol. 50, 231–241. 10.3109/10409238.2015.1023252 25788028 PMC4673598

[B7] Brunger A. T. Weninger K. Bowen M. Chu S. (2009). Single-molecule studies of the neuronal SNARE fusion machinery. Annu. Rev. Biochem. 78, 903–928. 10.1146/annurev.biochem.77.070306.103621 19489736 PMC2854664

[B8] Burre J. Sharma M. Tsetsenis T. Buchman V. Etherton M. R. Sudhof T. C. (2010). Alpha-synuclein promotes SNARE-complex assembly *in vivo* and *in vitro*. Science 329, 1663–1667. 10.1126/science.1195227 20798282 PMC3235365

[B9] Burre J. Vivona S. Diao J. Sharma M. Brunger A. T. Sudhof T. C. (2013). Properties of native brain alpha-synuclein. Nature 498, E4–E6; discussion E6–E7. 10.1038/nature12125 23765500 PMC4255827

[B10] Caughey B. Lansbury P. T. (2003). Protofibrils, pores, fibrils, and neurodegeneration: separating the responsible protein aggregates from the innocent bystanders. Annu. Rev. Neurosci. 26, 267–298. 10.1146/annurev.neuro.26.010302.081142 12704221

[B11] Chamberlain L. H. Burgoyne R. D. (1997). The molecular chaperone function of the secretory vesicle cysteine string proteins. J. Biol. Chem. 272, 31420–31426. 10.1074/jbc.272.50.31420 9395474

[B12] Chandra S. Gallardo G. Fernandez-Chacon R. Schluter O. M. Sudhof T. C. (2005). Alpha-synuclein cooperates with CSPalpha in preventing neurodegeneration. Cell 123, 383–396. 10.1016/j.cell.2005.09.028 16269331

[B13] Chernomordik L. V. Kozlov M. M. (2008). Mechanics of membrane fusion. Nat. Struct. Mol. Biol. 15, 675–683. 10.1038/nsmb.1455 18596814 PMC2548310

[B14] Choi B. K. Choi M. G. Kim J. Y. Yang Y. Lai Y. Kweon D. H.. (2013). Large alpha-synuclein oligomers inhibit neuronal SNARE-mediated vesicle docking. Proc. Natl. Acad. Sci. U.S.A. 110, 4087–4092. 10.1073/pnas.1218424110 23431141 PMC3593925

[B15] Choi B. K. Kim J. Y. Cha M. Y. Mook-Jung I. Shin Y. K. Lee N. K. (2015). Beta-Amyloid and alpha-synuclein cooperate to block SNARE-dependent vesicle fusion. Biochemistry 54, 1831–1840. 10.1021/acs.biochem.5b00087 25714795 PMC4414064

[B16] Crawford R. Torella J. P. Aigrain L. Plochowietz A. Gryte K. Uphoff S.. (2013). Long-lived intracellular single-molecule fluorescence using electroporated molecules. Biophys. J. 105, 2439–2450. 10.1016/j.bpj.2013.09.057 24314075 PMC3853080

[B17] Diao J. Burre J. Vivona S. Cipriano D. J. Sharma M. Kyoung M.. (2013a). Native alpha-synuclein induces clustering of synaptic-vesicle mimics via binding to phospholipids and synaptobrevin-2/VAMP2. Elife 2:e00592. 10.7554/eLife.00592 23638301 PMC3639508

[B18] Diao J. Ishitsuka Y. Lee H. Joo C. Su Z. Syed S.. (2012). A single vesicle-vesicle fusion assay for in vitro studies of SNAREs and accessory proteins. Nat. Protoc. 7, 921–934. 10.1038/nprot.2012.020 22582418 PMC4410872

[B19] Diao J. Yoon T. Y. Su Z. Shin Y. K. Ha T. (2009). C2AB: a molecular glue for lipid vesicles with a negatively charged surface. Langmuir 25, 7177–7180. 10.1021/la901676e 19563216 PMC2730783

[B20] Diao J. Zhao M. Zhang Y. Kyoung M. Brunger A. T. (2013b). Studying protein-reconstituted proteoliposome fusion with content indicators *in vitro*. Bioessays 35, 658–665. 10.1002/bies.201300010 23625805 PMC4453005

[B21] Fernandez-Chacon R. Wolfel M. Nishimune H. Tabares L. Schmitz F. Castellano-Munoz M.. (2004). The synaptic vesicle protein CSP alpha prevents presynaptic degeneration. Neuron 42, 237–251. 10.1016/S0896-6273(04)00190-4 15091340

[B22] Garcia-Junco-Clemente P. Cantero G. Gomez-Sanchez L. Linares-Clemente P. Martinez-Lopez J. A. Lujan R.. (2010). Cysteine string protein-alpha prevents activity-dependent degeneration in GABAergic synapses. J. Neurosci. 30, 7377–7391. 10.1523/JNEUROSCI.0924-10.2010 20505105 PMC6632397

[B23] Garcia-Reitbock P. Anichtchik O. Bellucci A. Iovino M. Ballini C. Fineberg E.. (2010). SNARE protein redistribution and synaptic failure in a transgenic mouse model of Parkinson's disease. Brain 133(Pt 7), 2032–2044. 10.1093/brain/awq132 20534649 PMC2892942

[B24] Hogen T. Levin J. Schmidt F. Caruana M. Vassallo N. Kretzschmar H.. (2012). Two different binding modes of alpha-synuclein to lipid vesicles depending on its aggregation state. Biophys. J. 102, 1646–1655. 10.1016/j.bpj.2012.01.059 22500765 PMC3318129

[B25] Hu R. Diao J. Li J. Tang Z. Li X. Leitz J.. (2016). Intrinsic and membrane-facilitated alpha-synuclein oligomerization revealed by label-free detection through solid-state nanopores. Sci. Rep. 6:20776. 10.1038/srep20776 26865505 PMC4749980

[B26] Jahn R. Lang T. Sudhof T. C. (2003). Membrane fusion. Cell 112, 519–533. 10.1016/S0092-8674(03)00112-0 12600315

[B27] Kaeser P. S. Regehr W. G. (2014). Molecular mechanisms for synchronous, asynchronous, and spontaneous neurotransmitter release. Annu. Rev. Physiol. 76, 333–363. 10.1146/annurev-physiol-021113-170338 24274737 PMC4503208

[B28] Larsen K. E. Schmitz Y. Troyer M. D. Mosharov E. Dietrich P. Quazi A. Z.. (2006). Alpha-synuclein overexpression in PC12 and chromaffin cells impairs catecholamine release by interfering with a late step in exocytosis. J. Neurosci. 26, 11915–11922. 10.1523/JNEUROSCI.3821-06.2006 17108165 PMC6674868

[B29] Nemani V. M. Lu W. Berge V. Nakamura K. Onoa B. Lee M. K.. (2010). Increased expression of alpha-synuclein reduces neurotransmitter release by inhibiting synaptic vesicle reclustering after endocytosis. Neuron 65, 66–79. 10.1016/j.neuron.2009.12.023 20152114 PMC3119527

[B30] Peethumnongsin E. Yang L. Kallhoff-Munoz V. Hu L. Takashima A. Pautler R. G.. (2010). Convergence of presenilin- and tau-mediated pathways on axonal trafficking and neuronal function. J. Neurosci. 30, 13409–13418. 10.1523/JNEUROSCI.1964-10.2010 20926667 PMC2962595

[B31] Russell C. L. Semerdjieva S. Empson R. M. Austen B. M. Beesley P. W. Alifragis P. (2012). Amyloid-beta acts as a regulator of neurotransmitter release disrupting the interaction between synaptophysin and VAMP2. PLoS ONE 7:e43201. 10.1371/journal.pone.0043201 22905234 PMC3419646

[B32] Sakon J. J. Weninger K. R. (2010). Detecting the conformation of individual proteins in live cells. Nat. Methods 7, 203–205. 10.1038/nmeth.1421 20118931 PMC2844853

[B33] Saura C. A. Choi S. Y. Beglopoulos V. Malkani S. Zhang D. Shankaranarayana Rao B. S.. (2004). Loss of presenilin function causes impairments of memory and synaptic plasticity followed by age-dependent neurodegeneration. Neuron 42, 23–36. 10.1016/S0896-6273(04)00182-5 15066262

[B34] Schindowski K. Belarbi K. Buee L. (2008). Neurotrophic factors in Alzheimer's disease: role of axonal transport. Genes Brain Behav. 7(Suppl. 1), 43–56. 10.1111/j.1601-183X.2007.00378.x 18184369 PMC2228393

[B35] Shankar G. M. Bloodgood B. L. Townsend M. Walsh D. M. Selkoe D. J. Sabatini B. L. (2007). Natural oligomers of the Alzheimer amyloid-beta protein induce reversible synapse loss by modulating an NMDA-type glutamate receptor-dependent signaling pathway. J. Neurosci. 27, 2866–2875. 10.1523/JNEUROSCI.4970-06.2007 17360908 PMC6672572

[B36] Sharma M. Burre J. Sudhof T. C. (2011). CSPalpha promotes SNARE-complex assembly by chaperoning SNAP-25 during synaptic activity. Nat. Cell Biol. 13, 30–39. 10.1038/ncb2131 21151134

[B37] Shen J. (2010). Impaired neurotransmitter release in Alzheimer's and Parkinson's diseases. Neurodegener. Dis. 7, 80–83. 10.1159/000285511 20173332 PMC2859234

[B38] Sudhof T. C. Rothman J. E. (2009). Membrane fusion: grappling with SNARE and SM proteins. Science 323, 474–477. 10.1126/science.1161748 19164740 PMC3736821

[B39] Terry R. D. Masliah E. Salmon D. P. Butters N. DeTeresa R. Hill R.. (1991). Physical basis of cognitive alterations in Alzheimer's disease: synapse loss is the major correlate of cognitive impairment. Ann. Neurol. 30, 572–580. 1789684 10.1002/ana.410300410

[B40] Tiwari S. S. d'Orange M. Troakes C. Shurovi B. N. Engmann O. Noble W.. (2015). Evidence that the presynaptic vesicle protein CSPalpha is a key player in synaptic degeneration and protection in Alzheimer's disease. Mol. Brain 8, 6. 10.1186/s13041-015-0096-z 25631211 PMC4314762

[B41] Wang C. Zhao C. Li D. Tian Z. Lai Y. Diao J.. (2016). Versatile structures of alpha-Synuclein. Front. Mol. Neurosci. 9:48. 10.3389/fnmol.2016.00048 27378848 PMC4913103

[B42] Weninger K. Bowen M. E. Choi U. B. Chu S. Brunger A. T. (2008). Accessory proteins stabilize the acceptor complex for synaptobrevin, the 1:1 syntaxin/SNAP-25 complex. Structure 16, 308–320. 10.1016/j.str.2007.12.010 18275821 PMC2856644

[B43] Weninger K. Bowen M. E. Chu S. Brunger A. T. (2003). Single-molecule studies of SNARE complex assembly reveal parallel and antiparallel configurations. Proc. Natl. Acad. Sci. U.S.A. 100, 14800–14805. 10.1073/pnas.2036428100 14657376 PMC299806

[B44] Wickner W. Schekman R. (2008). Membrane fusion. Nat. Struct. Mol. Biol. 15, 658–664. 10.1038/nsmb.1451 18618939 PMC2488960

[B45] Wu L. G. Saggau P. (1997). Presynaptic inhibition of elicited neurotransmitter release. Trends Neurosci. 20, 204–212. 10.1016/S0166-2236(96)01015-6 9141196

[B46] Yang Y. Kim J. Kim H. Y. Ryoo N. Lee S. Kim Y.. (2015). Amyloid-beta oligomers may impair SNARE-Mediated exocytosis by direct binding to syntaxin 1a. Cell Rep. 12, 1244–1251. 10.1016/j.celrep.2015.07.044 26279571 PMC4955600

